# Conformational Flexibility in the CD81-Binding Site of the Hepatitis C Virus Glycoprotein E2

**DOI:** 10.3389/fimmu.2018.01396

**Published:** 2018-06-18

**Authors:** Luisa J. Ströh, Kumar Nagarathinam, Thomas Krey

**Affiliations:** Institute of Virology, Hannover Medical School, Hannover, Germany

**Keywords:** hepatitis C virus, glycoprotein E2, neutralizing antibodies, conformational flexibility, immunoglobulin-like domain, CD81-binding site, vaccine design

## Abstract

Numerous antibodies have been described that potently neutralize a broad range of hepatitis C virus (HCV) isolates and the majority of these antibodies target the binding site for the cellular receptor CD81 within the major HCV glycoprotein E2. A detailed understanding of the major antigenic determinants is crucial for the design of an efficient vaccine that elicits high levels of such antibodies. In the past 6 years, structural studies have shed additional light on the way the host’s humoral immune system recognizes neutralization epitopes within the HCV glycoproteins. One of the most striking findings from these studies is that the same segments of the E2 polypeptide chain induce antibodies targeting distinct antigen conformations. This was demonstrated by several crystal structures of identical polypeptide segments bound to different antibodies, highlighting an unanticipated intrinsic structural flexibility that allows binding of antibodies with distinct paratope shapes following an “induced-fit” mechanism. This unprecedented flexibility extends to the entire binding site for the cellular receptor CD81, underlining the importance of dynamic analyses to understand (1) the interplay between HCV and the humoral immune system and (2) the relevance of this structural flexibility for virus entry. This review summarizes the current understanding how neutralizing antibodies target structurally flexible epitopes. We focus on differences and common features of the reported structures and discuss the implications of the observed structural flexibility for the viral replication cycle, the full scope of the interplay between the virus and the host immune system and—most importantly—informed vaccine design.

## Introduction

Approximately 71 million people worldwide are chronically infected with hepatitis C virus (HCV), which is one of the major causes of liver cirrhosis, liver failure, and hepatocellular carcinoma (1). Small-molecule drugs targeting HCV proteins termed direct-acting antivirals achieve cure rates of >95% (2), but high treatment costs, lack of awareness about hepatitis C, the emergence of multi-drug resistant viruses, and the need to protect patients from re-infection indicate that a prophylactic vaccine is still urgently required. In most viral infections, neutralizing antibodies (nAbs) are in the first line of defense of the adaptive immune response. For HCV, rapid induction of nAbs along with a broadly reactive T-cell response correlates with spontaneous clearance during acute infection and several studies highlighted the role of humoral immunity for the control both in the acute and chronic phase of infection (3, 4).

The two glycoproteins E1 and E2 of HCV are the major targets for nAbs. In particular, the receptor-binding glycoprotein E2 contains major antigenic determinants of HCV, mostly overlapping with binding sites for cellular receptors, including scavenger receptor class B type 1 (SR-B1) (5), the low-density lipoprotein receptor (LDLr) (6), and the tetraspanin CD81 (7). In addition to an extensive disulfide bridge network involving 8 and 18 conserved cysteines in E1 and E2, respectively, both proteins are heavily glycosylated in their N-terminal ectodomains (8, 9). Glycans are important for protein folding and affect epitope presentation and/or accessibility (10). The C-terminal transmembrane domains of E1 and E2 are anchored in the lipid envelope and interact to form an E1E2 heterodimer in HCV particles that are associated with lipoproteins and therefore also termed “lipo-viro particles” (11). Moreover, an E1 trimer observed at the surface of cell culture-derived HCV (HCVcc) and pseudoparticles suggested the presence of E1E2 heterodimers assembled as heterohexameric complexes (12, 13). However, due to the lack of structural information, many features of the architecture and glycoprotein arrangement at the surface of infectious HCV particles remain elusive.

E2 contains four hypervariable regions (HVR) termed HVR1 (residues 384–410 in the prototype H77 sequence), HVR2 (residues 460–485) (14, 15), HVR3 (residues 431–466) (16), and the intergenotypic variable region (igVR, residues 570–580) (17). The fact that the HVR1 interacts with SR-B1 and LDLr during virus entry (5, 6) would *per se* render this segment an interesting target for nAbs. Indeed, the first described HCV neutralization epitope is localized in HVR1 (18). However, nAbs targeting the HVR1 tend to be mostly strain specific, making the HVR1 less interesting for vaccine design (19). Although viruses lacking the HVR1 infect chimpanzees (20) they are more susceptible to neutralization by patient sera and other human mAbs (21–24), indicating that the HVR1 masks neutralization epitopes and serves as an “immune decoy,” recombinant glycoproteins lacking the HVR1 are not superior vaccine antigens (25). In addition, the binding of poorly neutralizing Abs to HVR1 can block the binding of broadly neutralizing Abs (bnAbs) to adjacent, conserved regions on E2 (26). These observed antagonistic effects suggest that the induction of anti-HVR1 Abs can interfere with a protective humoral response against HCV infection. By contrast, both HVR2 and the igVR seem neither to be direct targets for nAbs nor be directly involved in receptor binding. Nevertheless, similar to HVR1, both regions were found to modulate the accessibility of the CD81-binding site and the presentation of neutralizing epitopes on the E2 ectodomain (17, 27).

## Neutralization Epitopes

On the quest to develop a safe and efficient B-cell vaccine, numerous neutralization epitopes within the HCV glycoproteins have been mapped using a variety of approaches. Peptide scanning approaches using overlapping peptide libraries or random peptide display libraries have revealed a number of linear epitopes, but such an approach is not suitable to identify residues that contribute to conformation-sensitive epitopes (27). Another powerful approach is alanine scanning, probing panels of protein variants with distinct amino acid substitutions for binding to the Abs of interest (28–33). However, amino acid substitution frequently results in protein misfolding and thereby in false contact residues in case of conformation-sensitive epitopes—as illustrated for the bnAb AR3C, where the crystal structure revealed different contact residues than expected from previous alanine scanning (34). This pitfall is often alleviated by the use of non-competing conformational Abs to probe overall protein conformation and cross-competition analysis using a panel of well-characterized nAbs. *In vitro* studies of antibody escape can provide or confirm information about key epitopes (35–43). The gold standard to identify neutralization epitopes still remains the structural analysis of the immune complex, however, HCV glycoproteins are difficult to crystallize and only one neutralization epitope has been structurally characterized in complex with the E2 ectodomain to date (34). The combination of peptide and alanine scanning together with Ab cross-competition studies have yielded different nomenclature systems to describe and cluster epitopes on E2 to date such as antigenic domain A–E (44), antigenic region 1–5 (45), and epitope I–III (46). Of note, extensive overlap exists between these three systems of epitope nomenclature (47).

E1 is less immunogenic but two regions targeted by nAbs have been identified: residues 192–202 (in the prototype H77 sequence), which are recognized by the weakly nAb H-111 (48) and residues 313–324, which interact with the cross-reactive nAbs IGH-526 and IGH-505 (49, 50).

## E2 Structure and Conformational Flexibility

The two crystal structures of E2 core fragments, one in complex with the non-nAb 2A12 and the other with the bnAb AR3C, show that E2 features a central immunoglobulin (Ig)-like β-sandwich with two adjacent layers, one in front and one at the back (34, 51). Several regions of the protein are found in loop configurations or are disordered suggesting a high flexibility in parts of the structure (34). The igVR forms a disulfide-constrained loop within a flexible region spanning residues 567–596 but HVR1 and HVR2 are not included in the expression construct (51) or are only partially resolved in the electron density (34). Both structures are highly similar in the overall fold but the disulfide bond connectivity differs, suggesting that E2 features an enhanced plasticity compared to other viral glycoproteins, allowing for rather drastic local structural changes without affecting the overall fold of E2 (52). Of note, free thiol groups within the viral glycoproteins are required for virus entry (53), indicating a functional role of the observed plasticity.

**Figure 1:**
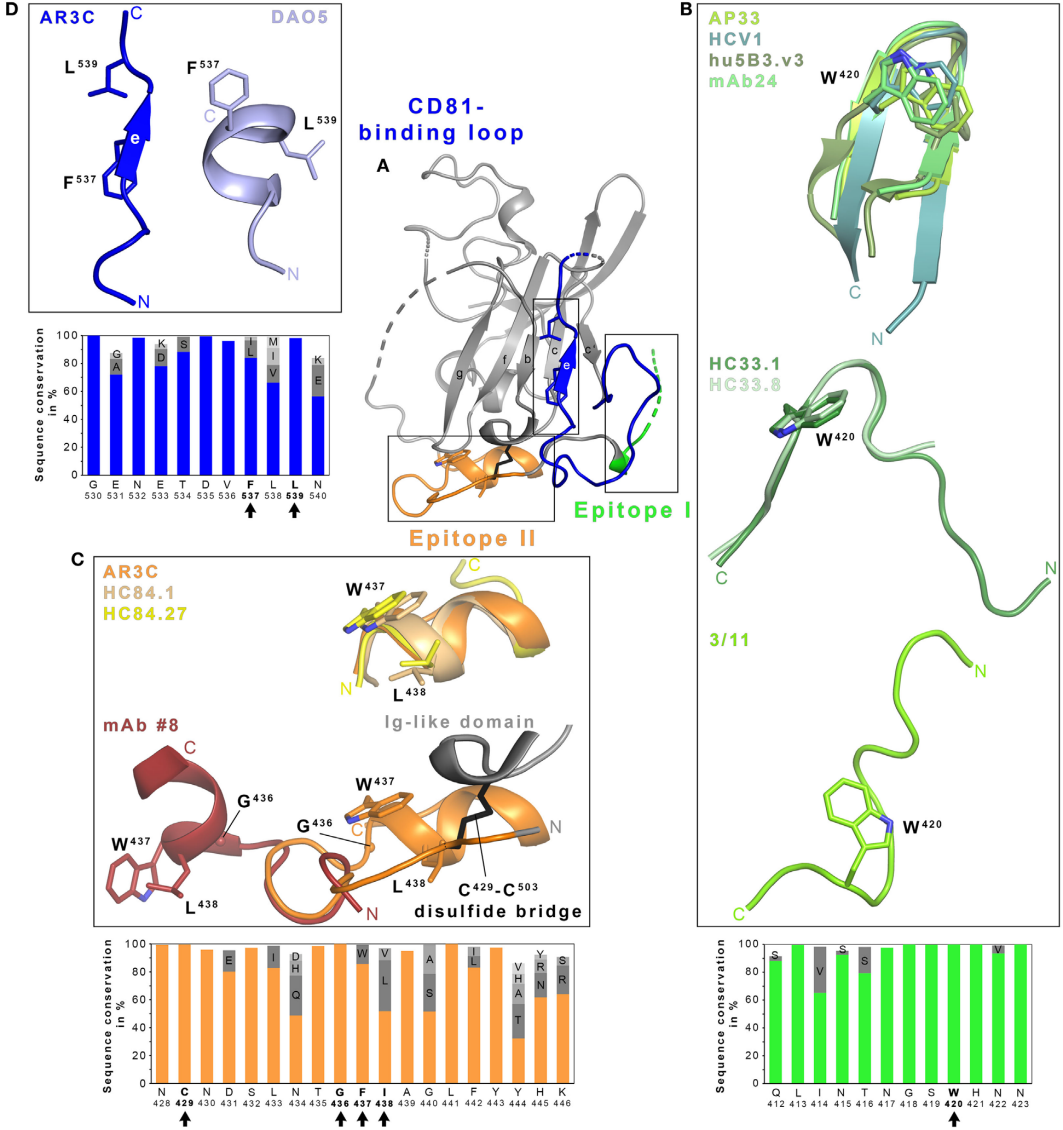
Structural flexibility of the hepatitis C virus (HCV) E2 glycoprotein. **(A)** Cartoon representation of the E2 ectodomain crystallized in complex with AR3C Fab (PDB 4MWF). The composite CD81-binding site consisting of epitope I (aa412–423; green), epitope II (aa428–446; orange), and the CD81-binding loop (aa518–542; blue) is highlighted in color and sidechains of selected residues are displayed as sticks. **(B–D)** Close-up views of the three antigenic sites mentioned above. **(B)** Epitope I is disordered in the context of the E2 structure but a synthetic epitope peptide folds as β-hairpin in complex with neutralizing antibodies (nAbs) AP33 (PDB 4GAJ), HCV1 (PDB 4DGV), hu5B3.v3 (PDB 4HS8), and mAb24 (PDB 5VXR) (upper panel). By contrast, the same peptide adopts two distinct extended conformations in complex with nAbs HC33.1 (PDB 4XVJ) and HC33.8 (PDB 5FGC) (middle panel) or 3/11 (PDB 4WHY; bottom panel), respectively. The peptide in the nAb HC33.4 complex (PDB 5FGB) adopts an amino acid backbone conformation identical to the one in complex with nAb HC33.8 and is not shown for simplicity. **(C)** Superposition of the epitope II peptide structure in complex with HC84.1 and HC84.27 Fabs (PDB 4JZN and 4JZO, respectively) onto the E2 ectodomain structure (upper panel) reveals a conserved 1.5-turn α-helix (aa437–442) with an extended C-terminal segment containing aa443–446. Superposition of the N-terminal loop of epitope II (aa430–434) from the peptide structure in complex with mAb #8 (PDB 4HZL) onto its counterparts in the E2 structure suggests that the short α-helix flips out to expose residues W^437^ and L^438^ for mAb #8 binding (bottom panel). **(D)** Residues 532–540 of the CD81-binding loop were observed in an extended conformation in the context of the E2 ectodomain structure **(A)** and in a helical conformation in the DAO5 Fab–E2 peptide complex structure (PDB 5NPJ) suggesting thereby a putative open and closed conformation of the immunoglobulin-like domain. Amino acid sequence conservation of the respective antigenic site was calculated across the six HCV genotypes for 481 isolate sequences (100 sequences each for genotypes 1, 2, 3, 6, and 70, 9 and 2 sequences for genotypes 4, 5, and 7, respectively) obtained and analyzed from the ViPR database (http://www.viprbrc.org) and is shown below each close-up view. Residues with side chains shown as sticks are highlighted by black arrows.

To date, no detailed structural information on the CD81–E2 interaction is available but different techniques including alanine scanning mutagenesis, negative stain electron microscopy, nAb competition experiments, and *in silico* docking have been applied to map the CD81-binding site on E2 (34, 54, 55). Critical contact residues include highly conserved residues W^420^, Y^527^, W^529^, G^530^, D^535^ (54), and the G^436^WLAGLF motif (55, 56) most of which are located within (1) a conserved N-terminal region (aa412–423), (2) a front layer region (aa428–446), and (3) an adjacent loop named CD81-binding loop (aa518–542). The majority of α-E2 bnAbs identified to date compete with CD81 for binding to E2. Hence, it is not surprising that their epitopes overlap with one or more of these three regions corresponding to three antigenic regions named epitope I, II, and III (46) (Figure 1). Interestingly, not all Abs targeting one of these three epitopes neutralize HCV infection, in spite of similar contact residues (57–59). For non-nAbs directed against epitope II interference with neutralization by nAbs targeting epitope I was proposed (60), but also cooperativity effects between nAbs directed against epitope I and nAbs targeting epitope II have been reported (29).

Within the last years a number of crystallographic studies have revealed molecular details of how Abs interact with these three epitopes, illustrating a great structural heterogeneity in particular within the epitope I (26, 41, 61–66), but also epitope II (59, 67–69), and more recently epitope III comprising the CD81-binding loop and parts of the core Ig-like domain (58). Of note, all three segments are largely conserved in sequence across all HCV genotypes and subtypes (Figure 1).

In the E2 core–AR3C Fab complex structure, epitope I is mostly disordered but synthetic peptides mimicking this epitope were complexed and crystallized with Fabs from bnAbs isolated from immunized rodents or from HCV-infected individuals. Human nAb HCV1, mouse nAbs AP33 and mAb24, and humanized and affinity-matured nAbs MRCT10.v362 and hu5B3.v3 (derived from AP33 and mu5B3, respectively) bind such epitope I peptides in a very similar β-hairpin conformation (Figure 1B) (41, 62, 63, 65, 66). However, the superposition of the linear epitope in complex with AP33 and HCV1 Fab reveal a 22° difference in the binding angle highlighting that both Abs engage the epitope on E2 from different directions (62). nAb paratopes are similar in shape and surface charge but interactions with E2 are realized by different nAb residues resulting in small conformational differences within a conserved β-hairpin conformation. In both cases, residues L^413^, N^415^, G^418^, and W^420^ of E2 are deeply buried in Fab-peptide interface.

By contrast, the same peptide is recognized in an extended conformation in a deep cleft between heavy and light chains of the Fab from the rat nAb 3/11 (Figure 1B) (61). E2 residues N^415^, W^420^, and H^421^ are especially critical for the 3/11-antigen interaction in line with epitope mapping by alanine scanning mutagenesis (32, 61). A third conformation of epitope I is recognized by a group of human mAbs named HC33 that were isolated from HCV-infected blood donors (Figure 1B) (64). In complex with the HC33.1 Fab, residues I^414^ and N^415^ form an anti-parallel β-sheet with strand F of the heavy chain variable region Ig domain and the remaining part of the peptide is recognized in an extended coil conformation. This interaction mode results in a turn (residues T^416^–S^419^) superimposing with the turn observed in the β-hairpin conformation in complex with HCV1 and AP33 Fabs. Residues L^413^, G^418^, and W^420^ constitute key anchors for the interaction and are deeply buried in the HC33.1-E2 interface (29, 64). An adaptive mutation N^417^S is associated with a shift of an N-linked glycosylation site from N^417^ to N^415^ that abolishes neutralization by nAbs HCV1, AP33, and mAb24. Residue N^415^ is buried by HCV1, AP33, and mAb24 but it is solvent-exposed in the HC33.1 Fab-peptide complex structure and allowing for glycosylation at N^415^ (29, 41, 64). Mutations N^417^S and N^415^D enhance the sensitivity to HC33.1 neutralization but also to neutralization by other human nAbs targeting different epitopes, suggesting that this region has a global impact on the conformation of HCV glycoproteins (41).

In the complex structures of the related HC33.4 and HC33.8 Fabs, a similar extended conformation is observed for E2 aa418–423 and aa415–423, respectively (26) (Figure 1B), but N-terminal residues aa412–414 are disordered. Although the HVR1-residue K^408^ was identified by alanine scanning mutagenesis to be part of the HC33.8- and HC33.4- but not of the HC33.1-epitope, no structural evidence for further epitope–paratope interactions beyond epitope I was observed (26). In summary, epitope I adopts at least three distinct conformations and greatly differs in its nAb interactions depending on the individual nAb. However, in all cases, the hydrophobic interaction networks involves W^420^, which is strictly conserved across HCV genotypes (Figure 1B) and serves also as a critical residue for CD81 binding (54). A recent electron microscopy study demonstrated that the HCV1 Fab binds soluble E2 from different angles of approach thereby further highlighting the conformational flexibility in epitope I (70).

At the surface of HCV particles, the epitope is either present in different conformations or readily converts between them (i.e., with a minimal kinetic barrier for conversion) and individual nAbs bind the epitope with their particular conformational selectivity. Indeed, the dose-dependent neutralization of nAbs 3/11 and AP33 suggests that the different conformations are in a dynamic equilibrium and can be converted in either direction (61). Interestingly, *in silico* predictions of the peptide alone propose a β-hairpin similar to the one observed in complex with Fabs from HCV1, AP33, and mAb24 (64). Together with the fact that the β-hairpin was observed in the majority of Fab complex structures, this suggests that the β-hairpin represents a preferred, but extremely unstable conformation on the HCV particle that can be readily converted into different conformations following an “induced-fit” binding mode to the antibody. This is further supported by the reported differences in neutralization potency of nAbs targeting the three described epitope I conformations (39, 71). The observation that nAbs targeting this segment usually have a broad neutralization activity suggests that genotype-specific sequence variations do not dictate the predominant epitope I conformation, although neutralization efficiency may be modulated by these sequence variations (61). The observed structural flexibility could explain the limited immunogenicity to the epitope I observed in HCV-infected patients (72).

Similarly, Fab-peptide structures provided molecular insights into recognition of epitope II. Structural information is available for epitope II in complex with different Abs—potent human nAbs on the one hand and weakly and non-nAbs derived from immunization with synthetic peptides on the other hand. When recognized by potent nAbs HC84.1, HC84.27, and the affinity-maturated nAb HC84.26.5D, an E2 peptide comprising aa434–446 forms a short α-helix spanning residues W^437^–F^442^ with an extended conformation on the C-terminal side comprising residues 443–446 (68, 69). This short α-helix can also be found in the AR3C Fab–E2 complex structure (34). Two other crystal structures of murine Fabs from the non-nAb #12 and the weakly neutralizing mAb #8 reveal an epitope that is located few amino acids upstream, but also includes the short α-helix (59, 67). Of note, residues W^437^ and L^438^ crucial for binding of mAb #12 and #8 are not accessible in the AR3C Fab–E2 complex, suggesting that a conformational change exposing these two residues is required to allow E2 binding. In line with this observation, superposition of the respective peptide structures using the N-terminus, which should be anchored to the Ig-like domain *via* a disulfide bridge (C^429^–C^503^), reveals that the C-terminal α-helix has to flip out to allow for mAb #8 binding (Figure 1C). This flexibility has been attributed to the strictly conserved G^436^ constituting a hinge between N- and C-terminus of the polypeptide chain, thereby resulting in an open and a closed state of E2 that implies the different presentation of epitope II (59, 70). Potent nAbs HC84.1, HC84.26.5D, and HC84.27 recognize the closed state similar to AR3C, indicating that this represents the preferred state of E2 in the viral particle and the open state targeted by weakly or non-nAbs is less frequently observed on virus particles. However, minor differences in the spatial arrangement of the C-terminal part of epitope II (aa443–446) (69) suggests that additional local structural changes may also occur in the closed conformation (59, 69).

A detailed functional and structural analysis of the non-nAb DAO5 provided a glimpse onto conformational changes in the CD81-binding loop (epitope III) and the adjacent part of the Ig-like domain (58) (Figure 1D). In the AR3C–E2 complex structure, the CD81-binding loop is stabilized by the Fab and the side chains of residues F^537^ and L^539^ (located on β-strand E) are buried inside the hydrophobic core of the Ig-like domain resembling a hypothetical closed conformation (34). In the absence of stabilizing Fab interactions, residues 524–535 are disordered and F^537^ is solvent-exposed (51). The crystal structure of non-nAb DAO5 Fab in complex with the E2 peptide aa532–540 reveals a helical conformation in which residues F^537^ and L^539^ are buried in the Fab interface, suggesting that on E2 they need to be solvent exposed to allow for interaction with DAO5 in a putative open conformation. A high sequence conservation within this region suggests that the observed conformational flexibility in the Ig-like domain is an intrinsic feature of E2. Indeed, both conformations are present simultaneously on infectious particles; hence, it is tempting to speculate that the open conformation recognized by non-Ab DAO5 acts as an immunological decoy that distracts the humoral immune system from the relevant CD81-binding conformation (58).

In addition to the static crystal structures, representing snapshots of an apparently highly dynamic protein, solution-based studies such as hydrogen deuterium exchange mass spectrometry (HDXMS) and limited proteolysis help to characterize flexible regions in E2 (51, 70). HDXMS detects the deuterium incorporation into the backbone amides when proteins are exposed to deuterated solvent. The exchange rate depends on the conformational flexibility and accessibility of individual residues to the solvent (73). As expected, HDXMS data confirmed the high structural flexibility in the E2 front layer including the composite CD81-binding site overlapping with epitopes of most bnAbs (70). Moreover, HVR1, HVR2, and igVR are highly flexible and heterogeneous in presented conformations in addition to being hypervariable in sequence (70). Interestingly, despite its unique conformational flexibility E2 has a high thermal stability when compared to proteins from thermophilic organisms or other viral envelope proteins such as HIV-1 env or influenza hemagglutinin presumably also due to its dense disulfide bridge network (70).

## Conclusion and Future Perspectives

The conformational flexibility within HCV E2 extends to the entire composite CD81-binding site, which overlaps most of the conserved neutralization epitopes present in E2. This finding raises the question how such a conformational flexibility emerges during virus evolution? Which functional importance does this flexibility have—or in other words—which selective advantage does this flexibility provide for the virus?

One possible explanation could be that the observed conformations represent different stages during virus entry, where a number of changes in environmental conditions (e.g., receptor binding, endosomal acidification, or a putative conformational change to fuse viral and endosomal membrane) may require different glycoprotein conformations. However, all nAbs mentioned above targeting epitope I block CD81 binding, suggesting that the different epitope I conformations can be adopted upstream of receptor binding. To date, the epitope I conformation in complex with CD81 remains elusive, but a conformationally flexible surface could be required for receptor binding. It is estimated that ~30% of protein–protein interactions include disordered protein regions (74) and the region within the large extracellular loop of CD81 thought to interact with E2 was described to display marked conformational fluctuations (75, 76). Therefore, a conformationally flexible surface on the glycoprotein may be favorable to establish a highly specific receptor interaction *via* an ordered interface following an induced fit binding mechanism. Structural studies on E2 in complex with CD81 will be required to further address this hypothesis.

Another possible explanation could be that the observed conformational flexibility is required for a putative dynamic rearrangement at the virus surface during infection of the host cell, resulting in exposure of the conserved receptor-binding region in E2—similar to the structural dynamics or “virus breathing” described for the related flaviviruses [reviewed in Ref. (77)]. Such an “opening” rearrangement would be in line with the observed time- and temperature-modulated exposure of neutralization epitopes on HCV virions (78).

A third possible explanation could be that this flexibility constitutes a viral mechanism to efficiently evade from nAbs. In general, the stability of peptides has been reported to directly correlate with their capacity to induce a humoral immune response (79), suggesting that conformational flexibility implies a modest immunogenicity. In line with this finding, immunization with a synthetic HCV epitope I peptide did not elicit bnAbs, likely due to its intrinsic structural flexibility (80) and several studies reported that even a cyclic variant of epitope I does not elicit high titers of Abs neutralizing HCV infection (80, 81).

This has important implications for vaccine design, suggesting that— although many subunit vaccine candidates based on HCV glycoproteins are currently under development—an unmodified form of the latter is limited in its capacity to elicit nAbs. Structure-guided stabilization of neutralization epitopes within E2 toward the conformation targeted by nAbs can potentially improve its immunogenic properties. Alternatively, a recent innovative approach termed epitope-focused vaccine design (82, 83) facilitates the design of epitope-specific immunogens to elicit nAbs where conventional vaccines failed to raise an immune response. For this purpose, a structurally characterized neutralization epitope is grafted onto an unrelated protein scaffold containing a segment with an identical backbone conformation. A successful example of this strategy is the development of an epitope scaffold presenting a single neutralization epitope of the human respiratory syncytial virus F protein and its neutralization potency can potentially be further augmented by the incorporation of further neutralization epitopes (82). Epitope-focused design has also been applied to HCV neutralization epitopes (80, 84) albeit with limited success. However, more recently an anti-idiotypic Ab, which also functions by mimicking a neutralization epitope on an unrelated protein (in this case an antibody), was demonstrated to robustly induce HCVcc-nAbs (85), suggesting that epitope-focused immunogens represent a viable strategy to develop a safe and efficient B cell vaccine and elicit a protective nAb response.

## Author Contributions

LS, KN, and TK participated in the design and coordination of the manuscript. All authors wrote, read, and approved the final version of the manuscript.

## Conflict of Interest Statement

The authors declare that the research was conducted in the absence of any commercial or financial relationships that could be construed as a potential conflict of interest. The handling Editor declared a past co-authorship with one of the authors [TK].
